# Bridging geographical disparities across 368 townships with healthcare system and socioeconomic factors in Taiwan

**DOI:** 10.1038/s41598-023-42124-y

**Published:** 2023-09-11

**Authors:** Chia-Ling Hsieh, Chia-Yu Chung, Hsin-Yu Chen, Shwn-Huey Shieh, Ming-Shun Hsieh, Vivian Chia-Rong Hsieh

**Affiliations:** 1 Department of Health Services Administration, China Medical University, No. 100, Sec. 1, Jingmao Road, Beitun District, Taichung, 406 Taiwan; 2 Department of Nursing, China Medical University Hospital, Taichung, Taiwan; 3 Department of Emergency Medicine, Taipei Veterans General Hospital, Taoyuan Branch, Taoyuan, Taiwan; 4 School of Medicine, National Yang Ming Chiao Tung University, Taipei, Taiwan; 5 Department of Emergency Medicine, Taipei Veterans General Hospital, Taipei, Taiwan

**Keywords:** Public health, Health policy

## Abstract

A universal health insurance program such as the National Health Insurance in Taiwan offers a wide coverage and increased access to healthcare services. Despite its ongoing efforts to enhance healthcare accessibility, differences in health for people living in urban and resource-deprived areas remain substantial. To investigate the longitudinal impact of the healthcare system and other potential structural drivers such as education and economic development on geographical disparities in health, we designed a panel study with longitudinal open secondary data, covering all 368 townships in Taiwan between 2013 and 2017. Our findings indicated higher mortality rates in the mountainous and rural areas near the east and south regions of the island in both years. Multivariate analyses showed an increase in the density of primary care physicians (PCP) was associated with lower all-cause mortality (*β* = − 0.72, *p* < 0.0001) and cardiovascular disease mortality (*β* = − 0.41, *p* < 0.0001). Effect of PCP is evident, but merely focusing on access to healthcare is still not enough. Additional measures are warranted to address the health disparities existing between urban and underprivileged areas.

## Introduction

For certain population groups, actual uptake of healthcare services can be often impeded by structural barriers despite universal availability of these resources within a health system. People living in different geographical areas may experience different levels of accessibility due to physical distances and barriers, which subsequently leads to geographical variation in health^1–3^. Numerous studies have highlighted the significant role of individual-level factors in shaping geographic inequalities^4,5^. Other studies, however, have shown that the influence from the nature of places, or the place’s structural elements, are much more important in our understanding of geographical variations in health. For example, a study suggested that regional healthcare markets, which are made up by predominantly groups of healthcare providers (supply), can generate significant geographic variation in healthcare utilization and spending after accounted for patient-level (demand) characteristics^6^. Juhn et al. (2021)^7^ examined COVID-19 epidemiology and found that community factors such as income level and living arrangements are significant factors of COVID-19 burden disparities. Other studies have also found significant impact of structural determinants of health on health equity such as education, employment, and other social and public policies^8,9^.

Taiwan is an island country with a population of roughly 23 million people and a land area of just slightly greater than Belgium (~ 36,000 square kilometers). It is divided into two sides by a central mountain range that spans the length of the country: the western terrain, where 90% of the population lives, is mostly flat and the eastern topography with mostly mountains and natural undeveloped landscape. There are five major geographic regions: north, center, south, east, and offshore archipelago islands which include Penghu, Matsu, and Kinmen. These regions consist of 368 cities/townships and districts in total, with six major municipalities that are directly governed by the central government. As in many other countries, bulk of the island’s population resides in the metropolitan areas, whereas most indigenous populations live in the mountainous and underserved areas.

Taiwan implemented a universal health insurance system in 1995. The National Health Insurance (NHI) is compulsory, single-payer social health insurance scheme that offers comprehensive benefits package to over 99% of the country’s population. Healthcare system like the NHI is an important determinant of health since they determine the types and quality of healthcare available to people^10^. The NHI has not only shown to enhance accessibility of healthcare services in different groups amongst the population^11^, but it has also effectively reduced the gap in life expectancy across socioeconomic groups^12^. Integrated Delivery System (IDS) was one of its programs enacted to further improve accessibility of healthcare in rural areas including the offshore islands. By having hospitals collaborating with local public health centers and primary care clinics, patients in rural and remote areas can gain regular access to primary care and dental care. A questionnaire study including 6400 respondents from various IDS programs concluded that both accessibility of services and the satisfaction level were both acceptable^13^. Yet, despite endeavors to improve healthcare access, significant and concerning health disparities persist between urban and resource-deprived areas^14,15^.

More importantly, no longitudinal proof of geographic health disparities at the township level exists, nor is there evidence of how changes in structural determinants of health over time affect population health in the long run. In this study, we divide this island country into 368 townships/cities and examine the change in their health outcomes between 2013 and 2017 in association with the healthcare system and other structural drivers.

## Methods

### Study design

We designed a panel study with longitudinal data of the 368 townships and cities in Taiwan using various sources of published national statistics in 2013 and 2017. Data sources included the Ministry of the Interior (population statistics and mortality), Ministry of Finance (income tax declaration and statistics), Ministry of Digital Affairs (education), and Ministry of Health and Welfare (main causes of death, cancer statistics, workforce and hospital capacity statistics), Taiwan. We selected years 2013 and 2017 to examine variable patterns over time, given they contained the most recent and the least amount of missing data across all sources. All data obtained were aggregated at the township-level and no one could not be identified individually, so informed consents were waived by the Institutional Review Board at the China Medical University Hospital (CRREC-109-103). All methods were performed in accordance with relevant guidelines and regulations.

### Measurements

#### Outcomes

Main outcome measures in this study were all-cause mortality (per 100,000), cardiovascular disease (CVD) mortality (per 100,000), and mortality attributable to unintentional injuries (UI) (per 100,000). All-cause mortality was computed from the total number of deaths from all causes (International Classification of Diseases, 10th Revision, Clinical Modification (ICD-10-CM): A00-Y98). CVD mortality was calculated using the number of deaths from heart-related diseases excluding hypertension (ICD-10-CM: I01-I02.0, I05-I09, I20-I25, I27, I30-I52). UI mortality accounted for the total number of deaths from accidents or unplanned events (ICD-10-CM: V01-X59, Y85-Y86). All outcome variables were measured at the township-level and age-standardized with the population’s age distribution. Our source of data was the Causes of Death by Township Statistics from the Ministry of Health and Welfare (MOHW), Taiwan. For UI deaths, misclassification bias is unlikely since the primary cause of death on the death registry is typically recorded based on the immediate cause of death. In the rare event of accidents or unplanned events, they should be appropriately recorded as the primary cause of death.

#### Healthcare system

To characterize the healthcare system at the township-level, we considered two main aspects under the NHI: workforce and institutional capacity. For workforce, primary care physician (PCP) density (per 100,000 population) was used to represent the availability of PC doctors in each township to offer generalist care. Density of specialists was not considered as statistics were categorized by medical specialty and a physician might be registered under multiple specialties, preventing an accurate estimate of specialists.

To sufficiently describe the institutional capacity of the hospitals, we used density of acute care (AC) beds (per 100,000 population), density of long-term care (LTC) beds (per 100,000 population), and density of intensive-care unit (ICU) beds (per 100,000 population). AC beds represent the number of hospital beds dedicated to short term care, not including those for mental conditions. LTC beds include the total number of hospital beds for chronic conditions, and ICU beds measure the number of hospital beds provided in intensive care wards, including neonatal ICUs. Although these three measures appear quite similar, they each capture distinct dimensions of a hospital's capacity for care delivery and are mutually exclusive.

#### Socioeconomic structure

Aside from healthcare system attributes, we included township-level structural determinants of health like education, economic development, and demographics. For education, we summed up the proportions of populations having achieved each level of education and applied the following equation to estimate the education level of each township according to the number of years in education (starting from elementary school up to postgraduate studies; where 29 is the maximum number of years of education that can be achieved assuming two years of masters studies and five years of doctoral studies on average). Thus, a higher calculated score would indicate higher educational achievement for each township/city.

$$\begin{aligned} {\text{Education}} = & \left[ {\left( {\#\, {\text{elementary graduates/total population}}} \right) * 12/29 + \left( {\# \,{\text{junior secondary}}/{\text{total population}}} \right)} \right. * 15/29 \\ & + \left( {\#\, {\text{senior secondary graduates / total population}}} \right) * 18/29 + \left( {\#\, {\text{undergraduates}}/{\text{total population}}} \right) * 22/29 \\ & \left. { + \left( {\#\, {\text{postgraduates}}/{\text{total population}}} \right) * 29/29} \right] * 100 \\ \end{aligned}$$

For the level of economic development, we ranked the 368 townships according to their annual median household income and categorized them into tertiles: low, middle, and high. Cut-off points were made at 33% (New Taiwan (NT) $66,680; ~ US$2,245) and 66% (NT$133,359; ~ US$4,490) using 2013 as the reference year. If the township annual median household income was ≤ NT$66,680, its economic development was classified as ‘low’; if it was between NT$66,681 and NT$133,358, its economic development was classified as ‘middle’; and if it was ≥ NT$133,359, then its economic development was classified as ‘high’. Income values were not adjusted since the inflation rate from 2013 to 2017 was acceptable at 2.92%.

#### Demographics

For the general demographics profile of each township, population density, sex ratio, and proportion of population aged 65 or over were measured for the two years of study. Population density was calculated by dividing population size by the geographical surface area (km^2^). Sex ratio was the number of males per 100 females. Proportion of population aged over 65 years was the population size aged 65 years or over divided by total population in each township. These demographic attributes were considered because they might exert confounding influence on our outcomes of interest.

### Data analysis

For 2013 and 2017, we calculated descriptive statistics for population health outcomes, healthcare system, education, economic development, and demographics in the 368 townships. Geospatial distribution of health outcomes was mapped across townships using QGIS 3.26 which is an open source geographic information system for geographical mapping. Due to the non-normality nature of the study variables, bivariate analyses were performed using Kruskal–Willis test to compare distributions of the three mortality rates across different economic levels for each year, while Mann–Whitney U test was conducted to compare the health indicators between the two years. Spearman correlation metric was used to examine correlations between all study variables. Effect size (ES) was estimated using two different tests: Cohen's D for continuous variables and Cramer's V for categorical variables.

Fixed effects regression model estimated the impact of healthcare system and structural determinants on the change in population health over time, from 2013 to 2017. Fixed effects model was chosen in order to avoid bias due to unobserved township-level factors that vary over time or can be correlated with study variables. In the multivariate analyses, we weighted the models by population density, proportion of population aged over 65, sex ratio, and adjusted for all predictors. Multicollinearity diagnostics were performed to confirm no predictor variables were collinear. All statistical analyses were performed using SAS statistical software package, version 9.4.

## Results

Table 1 shows the distribution of study variables for years 2013 and 2017. Of the three population health indicators, UI mortality showed the most obvious decline from 2013 to 2017 (ES: 0.0038), followed by all-cause mortality (ES: 0.0023), while CVD mortality marginally increased (ES: 0.0005), all of which were statistically insignificant (*p* > 0.05). Mean standardized all-cause mortality was 514.72 deaths per 100,000 in 2013 and 503.06 deaths per 100,000 in 2017; mean standardized CVD mortality was 56.73 deaths per 100,000 in 2013 and 57.20 deaths per 100,000 in 2017; and mean standardized UI mortality was 34.67 deaths per 100,000 in 2013 and 32.46 deaths per 100,000 in 2017.

**Table 1:** Distribution of study variables for 2013 and 2017.

	2013							2017							*p*	*Effect size*
	N	Mean n	SD %	Max	Min	Median	IQR	N	Mean n	SD %	Max	Min	Median	IQR		
Population health																
All-cause mortality	368	**514.72**	166.03	1414.70	172.00	478.00	108.94	368	**503.06**	145.01	1135.30	263.10	466.95	112.60	0.20	0.0023
CVD mortality	365	**56.73**	28.11	239.73	12.24	49.29	19.47	366	**57.20**	26.74	252.20	20.70	51.10	23.00	0.55	0.0005
UI mortality	341	**34.67**	21.07	162.09	6.10	29.89	22.40	360	**32.46**	20.27	136.60	4.70	28.15	19.60	0.10	0.0038
Healthcare system																
PCP density	368	**54.13**	43.08	486.79	0	46.00	37.42	368	**55.24**	39.51	277.87	0	47.51	41.32	0.57	0.0005
AC bed density	368	**314.55**	666.19	5966.14	0	0	346.20	368	**306.62**	665.61	6247.69	0	0	357.59	0.97	0
LTC bed density	368	**12.35**	62.96	829.13	0	0	0	368	**9.48**	53.50	819.58	0	0	0.00	0.68	0.0002
ICU bed density	368	**18.87**	46.26	463.67	0	0	17.85	368	**18.31**	46.08	453.82	0	0	19.30	0.96	0
Socioeconomic structure																
Education	368	**28.89**	4.12	89.63	21.63	28.65	2.71	368	**31.12**	3.99	82.91	23.35	31.03	2.98	< 0.01	0.1766
Economic development															0.96	0.0102
Low	–	**134**	36.41	–	–	–	–	–	**127**	34.51	–	–	–	–	–	–
Middle	–	**126**	34.24	–	–	–	–	–	**116**	31.52	–	–	–	–	–	–
High	–	**108**	29.35	–	–	–	–	–	**125**	33.97	–	–	–	–	–	–
Demographics																
Population density	368	**2775.76**	5662.00	40,089.00	5.00	643.00	1651.50	368	**2768.97**	5589.81	38,956.00	5.00	634.00	1721.00	0.93	0
Sex ratio	368	**107.05**	8.63	143.45	87.88	106.55	10.88	368	**106.29**	9.04	156.56	86.89	105.50	11.41	0.20	0.0022
Proportion of population aged 65 +	368	**13.75**	4.25	26.67	5.91	13.14	6.41	368	**15.81**	4.36	28.37	7.01	15.48	6.30	< 0.01	0.0524

*CVD* cardiovascular disease; *UI* unintentional injury; *PCP* primary care physician; *AC* acute care; *LTC* long-term care; *ICU* intensive-care unit.

*p*: p-value calculated using Mann–Whitney U test or Chi-square test; *Effect size*: Cohen’s D for continuous variables and Cramer's V for categorical variables.

For healthcare system, changes in all variables were also statistically insignificant. Mean PCP density increased from 54.13 physicians per 100,000 in 2013 to 55.24 physicians per 100,000 in 2017 (ES: 0.0005). Densities of AC, LTC, and ICU beds showed minimal changes.

Mean level of educational achievement improved significantly over the 4-year period, with an ES of 0.1766 (*p* < 0.01). Economic development level also shifted slightly towards the higher end, but without statistical significance (high economic development: 29.35% in 2013 and 33.97% in 2017, *p* = 0.9621).

**Figure 1:**
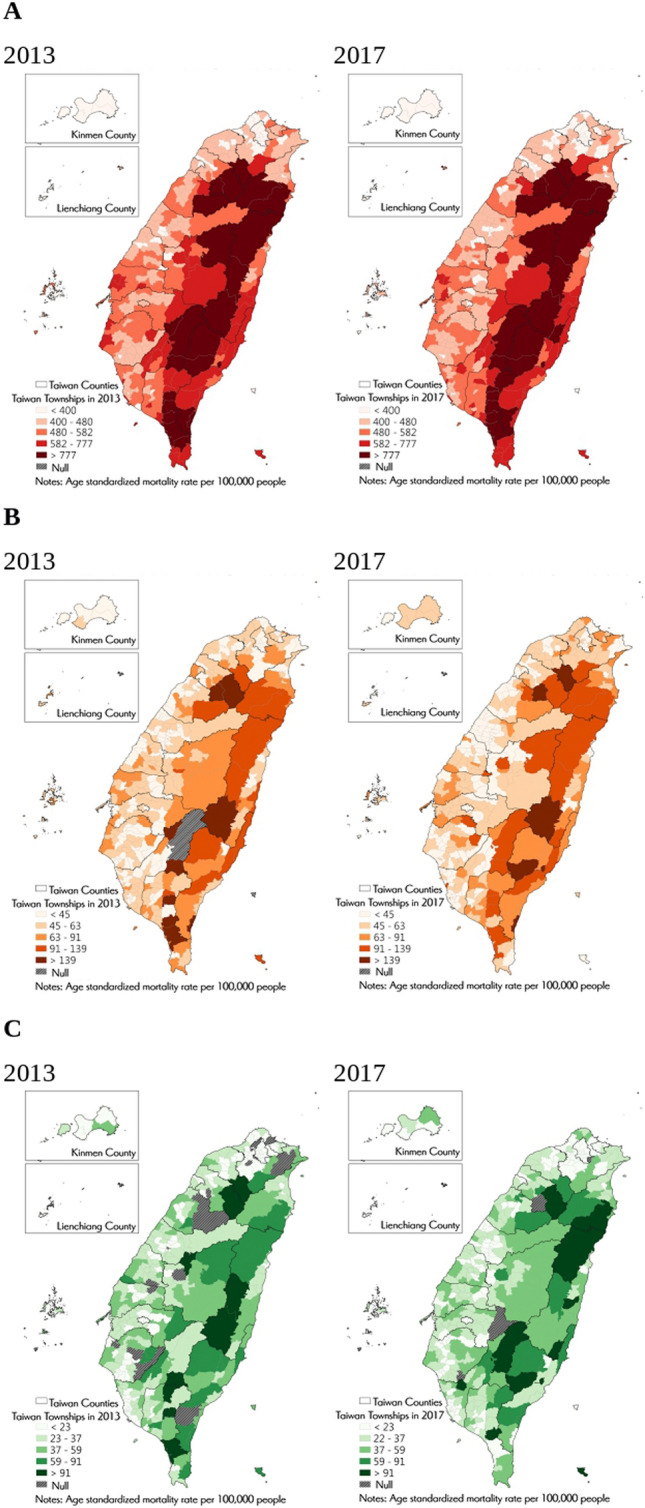
Geographical distribution of age-standardized mortality rates (all-cause, cardiovascular disease, unintentional injury) for 2013 and 2017 in Taiwan. Overall, higher mortality rates were found in the mountainous and rural areas near the east and south regions of the island in 2013 and 2017, such as Taoyuan District in Kaohsiung and Zhuoxi Township in Hualien. On the contrary, lowest mortality rates were detected in the metropolitan areas concentrated in the west and towards the country’s capital in Taipei. Note: (**A**) Age standardized all-cause mortality per 100,000 people. (**B**) Age standardized CVD mortality per 100,000 people. (**C**) Age standardized unintentional injury mortality per 100,000 people.

Figure 1 presents the geographical distribution of the three health indicators separately for 2013 (left panel) and 2017 (right panel). Although all-cause and UI mortality rates have generally decreased over the 4-year period, higher mortality rates were still detected in the mountainous and rural areas near the east and south regions of the island such as Taoyuan District in Kaohsiung and Zhuoxi Township in Hualien. Conversely, lowest mortality rates were found in the metropolitan areas concentrated in the west and towards the country's capital in the north, irrespective of time.

**Figure 2:**
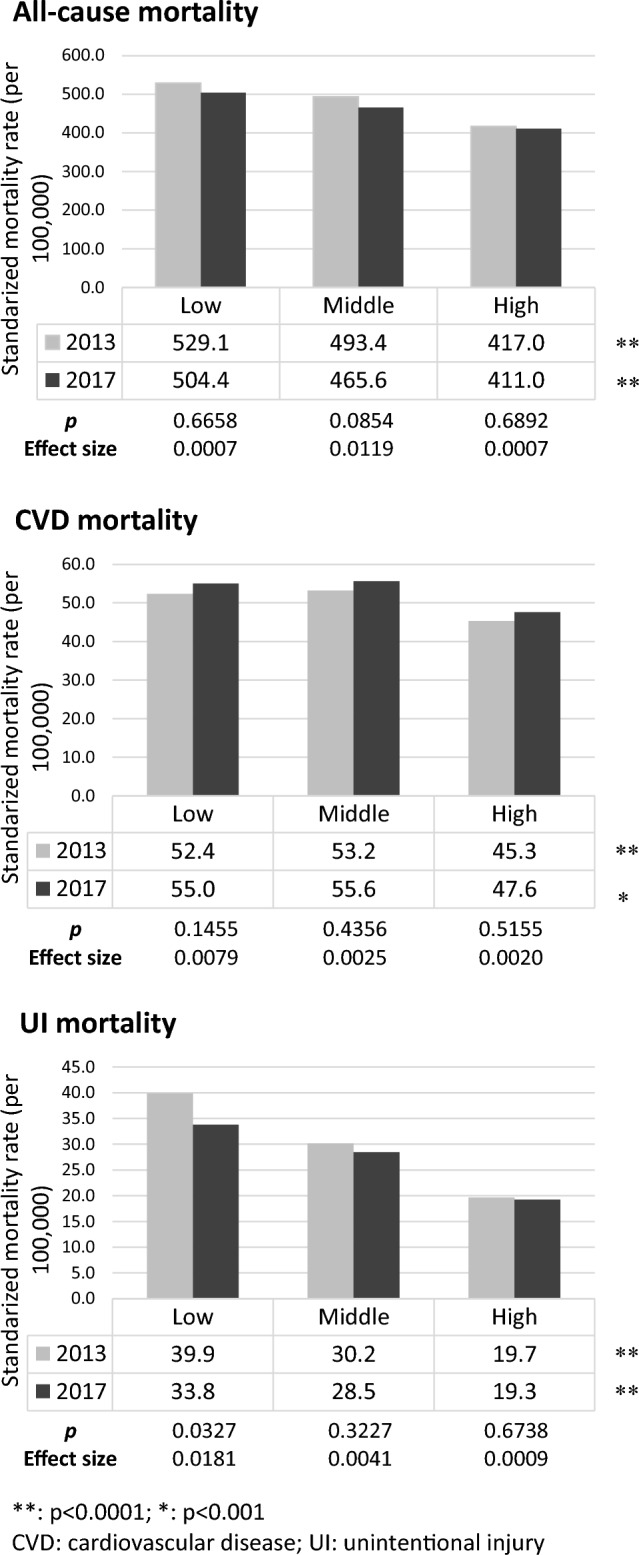
Population health comparison by township economic development level and year. There was significant difference across townships in all three mortality rates for 2013 and 2017. Townships with low economic development generally had the highest mortality rates. For all-cause and UI mortality, improvements were observed in the 4-year period especially for townships with low and middle economic development. For CVD mortality, although statistically insignificant, all townships experienced an increased mortality rate regardless of economic development level. **: p < 0.0001; *: p < 0.001. Note: CVD: cardiovascular disease; UI: unintentional injury.

When we stratify the townships according to their economic development, there was a significant difference (*p* < 0.0001) in all-cause mortality rates across different levels for 2013 and 2017 (Fig. 2). If we compare the indicator between 2013 and 2017 for each level, most substantial improvement was observed in townships with middle development, whose rate declined from 493.4 deaths per 100,000 in 2013 to 465.6 deaths per 100,000 in 2017, with an ES of 0.0119. For townships with high- and low-development levels, the decrease in all-cause mortality was to a lesser degree (ES: 0.0007). However, the declines were all statistically insignificant regardless of development level.

For CVD mortality, townships with high economic development exhibited significantly lower rates compared to both middle- and low-level townships (*p* < 0.0001 for 2013 and *p* < 0.001 for 2017) (Fig. 2). It is important to highlight that there was a rising (but statistically insignificant) trend in CVD mortality rate from 2013 to 2017, which was consistent across all townships irrespective of their level of economic development.

For UI mortality, a gradient emerged, with low-level economy townships showing the most significant decline and high-level economy townships displaying the least improvement: low-level (39.9 to 33.8 deaths per 100,000, ES: 0.0181, *p* = 0.0327), middle-level (30.2 to 28.5 deaths per 100,000, ES: 0.0041, *p* = 0.3227), and high-level (19.7 to 19.3 deaths per 100,000, ES: 0.0009, *p* = 0.6738). In both years, significant differences in UI mortality rates existed across townships of different economic development levels (*p* < 0.0001).

In Tables 2 and 3**,** strength of correlation between continuous explanatory variables and the three health outcomes is presented separately for 2013 and 2017. For both years, densities of PCP, AC bed, LTC bed, and ICU bed presented significant negative association with all mortality rates. AC bed density and ICU bed density seemed to have the strongest negative correlation with all three mortality rates, followed by PCP density and then LTC bed density. When examining socioeconomic influence, township's educational achievement achieved even stronger negative correlation with all three mortality rates compared to healthcare system variables in both years. This suggests that improving the population's educational attainment may help prevent mortality risks from CVD, UI, or all causes. Townships with higher sex ratios were observed with higher CVD, UI, and all-cause mortality rates.

**Table 2:** Correlation between continuous variables for year 2013.

	2013										
	All-cause mortality	CVD mortality	UI mortality	PCP density	AC bed density	LTC bed density	ICU bed density	Education	Population density	Sex ratio	Proportion of population aged 65 +
All-cause mortality	1	0.52***	0.69***	− 0.21***	− 0.39***	− 0.21***	− 0.37***	− 0.63***	− 0.64***	0.54***	0.21***
CVD mortality		1	0.34***	− 0.04	− 0.21***	− 0.09	− 0.19***	− 0.41***	− 0.38***	0.32***	0.02
UI mortality			1	− 0.31***	− 0.41***	− 0.23***	− 0.39***	− 0.60***	− 0.71***	0.63***	0.26***
PCP density				1	0.36***	0.19***	0.41***	0.31***	0.40***	− 0.50***	− 0.40***
AC bed density					1	0.45***	0.85***	0.42***	0.54***	− 0.54***	− 0.23***
LTC bed density						1	0.47***	0.22***	0.30***	− 0.30***	− 0.17***
ICU bed density							1	0.41***	0.53***	− 0.55***	− 0.25***
Education								1	0.66***	− 0.58***	− 0.21***
Population density									1	− 0.81***	− 0.43***
Sex ratio										1	0.56***
Proportion of population aged 65 +											1

*CVD* cardiovascular disease; *UI* unintentional injury; *PCP* primary care physician; *AC* acute care; *LTC* long-term care; *ICU* intensive-care unit.

Notes: Age standardized mortality rate per 100,000 people; *: *p* < 0.05; **: *p * < 0.01; ***: *p* < 0.001.

**Table 3:** Correlation between continuous variables for year 2017.

	2017										
	All-cause mortality	CVD mortality	UI mortality	PCP density	AC bed density	LTC bed density	ICU bed density	Education	Population density	Sex ratio	Proportion of population aged 65 +
All-cause mortality	1	0.46***	0.67***	− 0.24***	− 0.40***	− 0.20***	− 0.41***	− 0.68***	− 0.67***	0.59***	0.20***
CVD mortality		1	0.31***	− 0.16**	− 0.29***	− 0.15**	− 0.26***	− 0.42***	− 0.44***	0.35***	0.10
UI mortality			1	− 0.29***	− 0.35***	− 0.18***	− 0.39***	− 0.59***	− 0.66***	0.56***	0.21***
PCP density				1	0.38***	0.22***	0.44***	0.28***	0.41***	− 0.52***	− 0.33***
AC bed density					1	0.44***	0.85***	0.41***	0.55***	− 0.54***	− 0.19***
LTC bed density						1	0.47***	0.21***	0.30***	− 0.31***	− 0.14**
ICU bed density							1	0.41***	0.54***	− 0.58***	− 0.22***
Education								1	0.69***	− 0.57***	− 0.13*
Population density									1	− 0.80***	− 0.37***
Sex ratio										1	0.52***
Proportion of population aged 65 +											1

*CVD* cardiovascular disease; *UI* unintentional injury; *PC* primary care physician; *AC* acute care; *LTC* long-term care; *ICU* intensive-care unit.

Notes: Age standardized mortality rate per 100,000 people; *: *p* < 0.05; **: *p* < 0.01; ***: *p* < 0.001.

Table 4 shows the results from both crude and adjusted fixed effects models. When taking into account the change of township characteristics between 2013 and 2017, we see that an increase in PCP density was attributable to a decline in all-cause mortality (crude: *β* = − 0.72, *p* < 0.0001; adjusted: *β* = − 0.72, *p* < 0.0001) and CVD mortality (crude: *β* = − 0.41, *p* < 0.0001; adjusted: *β* = − 0.41, *p* < 0.0001) for the 368 townships. This indicates that, each unit increase in township's PCP density would cause its all-cause mortality and CVD mortality to fall by 0.72 and 0.41, respectively. Similarly, an increase in AC bed density would indicate a drop in UI mortality by 0.05 in the adjusted model (*β* = − 0.05, *p* = 0.01), while an increase in ICU bed density would have the opposite effect (*β* = 0.36, *p* = 0.005). The socioeconomic determinants, education or economic development, did not seem to exert a significant impact on any of the population health outcomes after adjusting for all covariates.

**Table 4:** Fixed effects model for population health disparities.

	All-cause mortality						CVD mortality						UI mortality					
	Crude			Adjusted*			Crude			Adjusted*			Crude			Adjusted*		
	β	SE	*p*	β	SE	*p*	β	SE	*p*	β	SE	*p*	β	SE	*p*	β	SE	*p*
Healthcare system																		
PCP density	**− 0.72**	0.13	< .0001	**− 0.72**	0.14	< .0001	**− 0.41**	0.05	< .0001	**− 0.41**	0.05	< .0001	**− 0.16**	0.12	0.17	**− 0.09**	0.12	0.49
AC bed density	**0.05**	0.03	0.08	**0.12**	0.07	0.13	**0.01**	0.01	0.30	**− 0.01**	0.03	0.62	**0.005**	0.01	0.43	**− 0.05**	0.02	0.01
LTC bed density	**− 0.07**	0.29	0.82	**− 0.20**	0.26	0.44	**− 0.10**	0.12	0.36	**− 0.13**	0.09	0.16	**0.04**	0.07	0.52	**0.06**	0.07	0.33
ICU bed density	**0.26**	0.18	0.15	**− 0.52**	0.50	0.30	**0.09**	0.07	0.21	**0.15**	0.18	0.38	**0.07**	0.04	0.08	**0.36**	0.13	0.005
Socioeconomic structure																		
Education	**0.10**	0.68	0.88	**− 0.02**	0.62	0.98	**− 0.20**	0.27	0.47	**− 0.30**	0.22	0.18	**0.21**	0.16	0.18	**0.14**	0.16	0.35
Economic development																		
Increase	**12.2**	11.0	0.27	**8.63**	10.1	0.39	**6.74**	4.44	0.13	**4.76**	3.61	0.19	**1.30**	2.56	0.61	**0.47**	2.59	0.86
Decrease	**− 15.1**	38.2	0.69	**− 26.9**	34.9	0.44	**23.0**	15.3	0.13	**− 21.1**	12.5	0.09	**− 6.95**	8.79	0.43	**− 4.18**	8.77	0.63
No change	**− 10.2**	10.7	0.34	**1.00**	–	–	**− 8.11**	4.27	0.06	**1.00**	–	–	**− 0.66**	2.47	0.79	**1.00**	–	–

*CVD* cardiovascular disease; *UI* unintentional injury; *PCP* primary care physician; *AC* acute care; *LTC* long-term care; *ICU* intensive-care unit; *SE* standard error.

*Model adjusted for sex ratio and proportion of population aged 65 +  and weighted by population density.

## Discussion

Through our panel study of geographical disparities in Taiwan, we discovered that population health had improved overall from 2013 to 2017. Within the 4-year period, greatest improvements in UI and all-cause mortality were generally seen in areas with low and middle economic development. Nevertheless, significant disparities in mortality rates between rural/mountainous regions and metropolitan townships remained. After controlling for population demographics, education, and economic development, we found that healthcare systems with persistent emphasis on PC generally yielded better health outcomes over time (i.e., decreased all-cause and CVD mortality rates), even at the local level.

Following the release of the Commission on Social Determinants of Health report in 2008, there has been a worldwide effort to tackle health inequality through the social determinants of health approach. Many countries, however, still face rising health inequalities despite their sustained improvements in health. In Europe, for example, a review of health disparities in the continent suggested obvious patterns of within-country geographical differences in health, including the UK, Spain, France, and Germany^16^. Similarly, a repeated cross-sectional study from Canada revealed worsening inequalities in self-reported health among employed and unemployed citizens between 2000 and 2014^17^. Studies from Asia provided similar findings^18,19^.

There are several possible factors contributing to the geographical disparities in Taiwan. As our findings indicate, access to primary care (PC) may be one of them. In her prominent series of work on PC, Professor Barbara Starfield highlighted the positive impact of PC on health and the effect of the supply of PCPs on reducing income-inequality on health^20–22^. This has also been the case for different geographical areas in Taiwan. By facilitating first-contact and people-centered care, in contrast to specialty care, PC places a greater focus on prevention and effectively increases access to health services for disadvantaged population groups^21^. A previous study of 102 countries found that expanded coverage of PC services is an affordable means that can be beneficial to population particularly in low and middle-income settings^23^. Evidence suggested that provision of PC in socially and economically deprived areas may decrease the likelihood of child mortality, potentially due to adequate referral mechanisms, continuity of care, and receiving PC in public facilities^24^.

Since PCP was found to be protective factor for all-cause mortality and CVD mortality in this study, but not UI mortality, it is possible to speculate that the former two health indicators included in part mortality amenable to healthcare. These are deaths from certain causes that are potentially preventable with timely and effective health care. This has often been used as a measure of health system performance in developed countries and includes deaths due to tuberculosis, diabetes mellitus, and ischemic heart disease^25^. Our evidence suggests the vital role of PCPs in sustaining the performance of Taiwan’s healthcare system.

It is essential to promote an equitable distribution of PCP services among townships. Nonetheless, the impact of PCP density on UI mortality did not appear to be significant. Other factors, such as modes of transportation, street planning, and pedestrian-friendly infrastructure, may play a more important role in preventing unforeseen accidents and injuries^26–28^. Unfortunately, these data were mostly not available at the township level. We did observe, however, a social gradient in the decline of UI mortality, i.e., the largest drop in UI mortality in low economic development townships, followed by middle and high economic development areas, respectively. While improved access to AC was linked to a reduction in UI mortality, we also noticed a higher incidence of UI mortality in areas with greater access to ICU facilities. This could be attributed to a higher number of critically ill patients receiving treatment in those areas. Furthermore, the reduction in UI and all-cause deaths may serve as contributing factors to the increased life expectancy during this period.

High economic development townships exhibited the lowest CVD mortality rate compared to middle and low development townships. There was, however, a slight increase in this health indicator across all townships from 2013 to 2017. Despite many high-income countries experiencing significant reductions in CVD deaths over the past decades, Taiwan, like some other high-income nations, is witnessing a slowdown in the rate of decline. This trend can be attributed to factors such as sedentary lifestyle and the increasing prevalence of obesity, diabetes, and hypertension^29,30^. From the 1980s to the 2000s, Taiwan experienced a significant reduction in CVD mortality. Nevertheless, dietary shifts among younger generations towards higher calorie intake and sedentary lifestyles led to health concerns like overweight and metabolic syndrome. This trend is further compounded by an aging population with a higher prevalence of comorbidities, all of which collectively contribute to the rising CVD mortality rates.

Our study has other limitations. We did not consider the impact of specialist clinicians due to several reasons; first, data availability of specialists practicing at the township level was scarce and; second, calculating physician density for each area was difficult due to the possibility of a physician being registered with two or more specialties. Also to factor in the contribution by employment and access to work, we used data on the economic development level of each township as a proxy. The economic development level was determined by ranking annual median household income of all 368 townships. We believe that annual median household income can serve as a good representation of people's employment opportunities and condition, because it reflects how families are earning; higher incomes would not only indicate access to work, but it would also suggest having jobs with better wages. Results from our correlation analyses indicated that sex ratio was positively associated with all three mortality endpoints, which suggests that areas with more males had more deaths from CVD, UI, and all-causes. A potential gender disparity could exist, but because demographics was not our main variable of interest, future investigation is needed to examine this association more closely. Lastly, although a period of four years for a longitudinal analysis may not seem extensive, we were able to demonstrate the existing geographical variations in health and their significant determinants during this period. Another longitudinal analysis may be feasible should more updated and comprehensive data becomes available in the near future.

Since the government's release of the Health Inequalities in Taiwan report in 2016^15^, which was led by Sir Michael Marmot and his team from University College London, a certain level of awareness had been raised about the health disparities among different social groups and their possible underlying factors. Unfortunately, it appears that the issue of health inequality is still being viewed through the lens of 'health care', rather than being approached from a broader scope, like ‘health in all policies’ (HiAP). Currently, there are incentives to recruit more junior and government-sponsored doctors to work in mountainous and rural areas. The Integrated Delivery System provides ongoing and routine specialist outpatient services in these areas^31^. Tertiary hospitals are also subsidized for allocating critical care and trauma personnel to these areas. Other related social and public policies include providing financial support for low-income households through social welfare schemes and programs aimed at preventing chronic diseases through regular public health screening^32^. While the positive impact of PCPs is evident, targeted interventions for disadvantaged areas are essential. We must acknowledge that health is shaped by individuals’ lifestyles, which are, in turn, shaped by the social and economic environments in which we live in, such as the conditions in which we are born, grow, live, work, and age^10,33^.

## Conclusions

Findings of this study highlight the potential of primary care access in promoting a more equitable distribution of health. Despite ongoing efforts to improve healthcare accessibility, our findings also underscore the persistence of health disparities between individuals residing in urban and resource-deprived areas over time. Further research and targeted interventions are warranted to bridge these gaps and ensure that all populations can access quality healthcare services and achieve improved health outcomes.

## Data Availability

VCH, CLH, CYC, and HYC had full access to the data and take responsibility for the integrity of the data and the accuracy of the data analysis. VCH is the guarantor. All data generated or analysed during this study are included in this published article (and its Supplementary Information files).

## References

[1] Burke M, Heft-Neal S, Bendavid E (2016). Sources of variation in under-5 mortality across sub-Saharan Africa: A spatial analysis. Lancet Glob. Health..

[2] Rosenberg B (2016). Quantifying geographic variation in health care outcomes in the United States before and after risk-adjustment. PLoS ONE.

[3] Chen CH (2006). Long-term trends and geographic variations in the survival of patients with hepatocellular carcinoma: Analysis of 11,312 patients in Taiwan. J. Gastroenterol. Hepatol..

[4] Baum A, Wisnivesky J, Basu S, Siu AL, Schwartz MD (2020). Association of geographic differences in prevalence of uncontrolled chronic conditions with changes in individuals' likelihood of uncontrolled chronic conditions. JAMA.

[5] Gabert R, Thomson B, Gakidou E, Roth G (2016). Identifying high-risk neighborhoods using electronic medical records: A population-based approach for targeting diabetes prevention and treatment interventions. PLoS ONE.

[6] Finkelstein A, Gentzkow M, Williams H (2016). Sources of geographic variation in health care: Evidence from patient migration. Q. J. Econ..

[7] Juhn YJ (2021). Role of geographical risk factors in COVID-19 epidemiology: Longitudinal geospatial analysis. Mayo Clin. Proc. Innov. Qual. Outcomes.

[8] Courtin E, Kim S, Song S, Yu W, Muennig P (2020). Can social policies improve health? A systematic review and meta-analysis of 38 randomized trials. Milbank Q..

[9] Osypuk TL, Joshi P, Geronimo K, Acevedo-Garcia D (2014). Do social and economic policies influence health? A review. Curr. Epidemiol. Rep..

[10] Commission on Social Determinants of Health (CSDH). Closing the gap in a generation: Health equity through action on the social determinants of health. (World Health Organization, Geneva, 2008).

[11] Cheng SH, Chiang TL (1997). The effect of universal health insurance on health care utilization in Taiwan. Results from a natural experiment. JAMA.

[12] Wen CP, Tsai SP, Chung WSI (2008). A 10-year experience with universal health insurance in Taiwan: Measuring changes in health and health disparity. Ann. Intern. Med..

[13] Lin W, Hsieh C, Hsu YE (2017). Consumers’ assessment on integrated delivery system in remote areas in Taiwan. Value Health.

[14] Hsu JC, Tseng YC, Chang SM, Lee YC, Lin PC, Chu HJ (2020). Health inequality: A longitudinal study on geographic variations in lung cancer incidence and mortality in Taiwan. BMC Public Health.

[15] Health Promotion Administration (HPA), Ministry of Health and Welfare, Taiwan and University College London (UCL), Institute of Health Equity. Health inequalities in Taiwan. (Taipei: Taiwan. 2016).

[16] Mackenbach JP, Karanikolos M, McKee M (2013). The unequal health of Europeans: Successes and failures of policies. Lancet.

[17] Shahidi FV, Muntaner C, Shankardass K, Quiñonez C, Siddiqi A (2018). Widening health inequalities between the employed and the unemployed: A decomposition of trends in Canada (2000–2014). PLoS ONE.

[18] Min JW (2014). Trends in income-related health inequalities in self-assessed health in Korea, 1998–2011. Glob. Public Health..

[19] Campostrini S, Dal Grande E, Taylor AW (2019). Increasing gaps in health inequalities related to non-communicable diseases in South Australia; implications towards behavioural risk factor surveillance systems to provide evidence for action. BMC Public Health.

[20] Shi L, Starfield B (2001). The effect of primary care physician supply and income inequality on mortality among blacks and whites in US metropolitan areas. Am. J. Public Health.

[21] Starfield B, Shi L, Macinko J (2005). Contribution of primary care to health systems and health. Milbank Q..

[22] Shi L, Starfield B, Politzer R, Regan J (2002). Primary care, self-rated health, and reductions in social disparities in health. Health Serv. Res..

[23] Hsieh VCR, Wu JCL, Wu TN, Chiang TL (2015). Universal coverage for primary health care is a wise investment: Evidence from 102 low- and middle-income countries. Asia Pac. J. Public Health.

[24] Reyes H, Perez-Cuevas R, Salmeron J, Tome P, Guiscafre H, Gutierrez G (1997). Infant mortality due to acute respiratory infections: The influence of primary care processes. Health Policy Plan..

[25] Nolte E, McKee M (2003). Measuring the health of nations: Analysis of mortality amenable to health care. BMJ.

[26] Lichtman-Sadot S (2019). Can public transportation reduce accidents? Evidence from the introduction of late-night buses in Israeli cities. Reg. Sci. Urban Econ..

[27] Mohan D, Bangdiwala SI, Villaveces A (2017). Urban street structure and traffic safety. J. Saf. Res..

[28] Schuurman N, Cinnamon J, Crooks VA, Hameed SM (2009). Pedestrian injury and the built environment: An environmental scan of hotspots. BMC Public Health.

[29] Lopez AD, Adair A (2019). Is the long-term decline in cardiovascular-disease mortality in high-income countries over? Evidence from national vital statistics. Int. J. Epidemiol..

[30] Health Promotion Administration Taiwan. National Cardiovascular Disease Prevention and Control Phase I Program (2018–2023). Health Promotion Administration, Ministry of Health and Welfare, Taiwan (2018) (Chinese).

[31] Chen LJ, Chang YJ, Shieh CF, Yu JH, Yang MC (2016). Accessibility of ophthalmic healthcare for residents of an offshore island-an example of integrated delivery system. BMC Health Serv. Res..

[32] Lin YH, Kao CC (2013). Factors influencing colorectal cancer screening in rural southern Taiwan. Cancer Nurs..

[33] Dahlgren, G. & Whitehead, M. Policies and strategies to promote social equity in health. (Institute for Futures Studies, Stockholm. 1991).

